# The relationship between survival rate and intradialytic blood pressure changes in maintenance hemodialysis patients

**DOI:** 10.1080/0886022X.2017.1305407

**Published:** 2017-03-24

**Authors:** Jiayue Lu, Minxia Zhu, Shang Liu, Mingli Zhu, Huihua Pang, Xinghui Lin, Zhaohui Ni, Jiaqi Qian, Hong Cai, Weiming Zhang

**Affiliations:** a Department of Nephrology, South Campus, Renji Hospital, School of Medicine, Shanghai Jiao Tong University, Shanghai, China;; b Department of Nephrology, Renji Hospital, School of Medicine, Shanghai Jiao Tong University, Shanghai, China

**Keywords:** Hemodialysis, blood pressure, hypertension, mortality

## Abstract

**Objective:** The objective of this study is to investigate the relationship between blood pressure changes and all-cause mortality, and between blood pressure changes and cardiovascular mortality, for maintenance hemodialysis (MHD) patients during dialysis.

**Methods:** Data regarding general condition, biochemical indices, and survival prognosis of MHD patients who were treated at the Shanghai Jiao Tong University School of Medicine-affiliated Renji Hospital from July 2007 to December 2012 were collected, in order to evaluate the relationship between patients’ blood pressure changes during hemodialysis and mortality.

**Results:** Among 364 patients, with an average age of 63.07 ± 13.93 years, an average dialysis vintage of 76.00 (range, 42.25–134.00) months, and a follow-up time of 54.86 ± 19.84 months, there were 85 cases (23.4%) of all-cause death and 46 cases (14.2%) of cardiovascular death. All-cause mortality and cardiovascular mortality were lowest (OR, 0.324 and 0.335; 95% CI, 0.152–0.692 and 0.123–0.911; *p* value, .004 and .032, respectively) in patients whose systolic blood pressure difference (ΔSBP) before and after dialysis was between 7.09 and 14.25 mmHg. Kaplan–Meier analysis indicated that both all-cause mortality and cardiovascular mortality were markedly increased for patients with ΔSBPless than −0.25 mmHg (*p* value, .001 and .044, respectively). Cox regression analysis showed that ΔSBP< −0.25 mmHg, hemoglobin concentration, *Kt/v * and albumin were independent risk factors for all-cause mortality in MHD patients.

**Conclusions:** MHD patients whose blood pressure increased significantly after hemodialysis had a higher risk of dying; ΔSBP, hemoglobin concentration, *Kt/v* and albumin were independent risk factors for all-cause mortality in MHD patients.

Mortality in maintenance hemodialysis (MHD) patients is high, especially cardiovascular mortality, which is 3–10 times higher than in the general population.^1^ The increase in mortality is closely related to cardiovascular risk factors. These include changes in blood pressure (BP) such as a decrease or marked increase in systolic blood pressure (SBP) before hemodialysis,^2^ hypotension occurring during hemodialysis, changes in the structure of the heart such as left ventricular hypertrophy,^3^ extracellular overhydration,^4^ or neural hormone imbalance.

Studies have shown that, for the non-dialysis population, blood pressure variability is a risk factor for cardiovascular events, stroke, and left ventricular hypertrophy.^5^ Similarly, blood pressure variability is one of the most important cardiovascular risk factors for MHD patients. There is some evidence that, for MHD patients, SBP variability during treatment is independent of average SBP value, and that it is a strong predictor of stroke and transient ischemic attack (TIA), even when antihypertensive drugs are used.^6^ It has been reported by a few studies that SBP increase before hemodialysis is an independent risk factor for all-cause mortality in MHD patients.^7^ However, there is a lack of data about the relationship between large differences in blood pressure before and after hemodialysis and long-term survival in MHD patients. In this study, a retrospective analysis was conducted involving MHD patients who had been treated at our hospital, in order to evaluate changes in blood pressure before and after dialysis, and to investigate the relationship between blood pressure variability during dialysis and all-cause mortality, as well as cardiovascular mortality.

## Subjects and methods

### Research subjects

Four hundred and two patients, who were registered in the Shanghai Dialysis Registration System and have received ≥3 months of MHD treatment at the Shanghai Jiao Tong University Medical School-affiliated Renji Hospital from 1 July 2007 to 31 December 2012, were selected. Thirty-eight of them were excluded due to incomplete registration data, and the remaining 364 patients were included in the study.

Patients’ demographic and clinical data were collected, including gender, age, height, dry weight, dialysis vintage, blood pressure before and after dialysis, biomedical indices, etc. All patients were treated with bicarbonate dialysis fluid and F80 (Fresenius Co., Homburg, Germany) or REXEED (Asahi Kasei Corp., Tokyo, Japan) polysulfone membrane dialyzer. The dialysis was conducted 2–3 times a week, lasting 4–5 h each time (10–12 h/week) with 200–350 ml/min blood flow. Filtration volume was exceeded every time, in order to reach clinical dry weight.

### Research methods

(1) Blood biochemical indices, including average values of corrected calcium (Ca^2+^), phosphate (P^3+^), intact parathyroid hormone (iPTH), total cholesterol (TC), triglyceride (TG), low-density lipoprotein (LDL) cholesterol, high-density lipoprotein (HDL), cholesterol, albumin (Alb), hemoglobin (Hb), and high-sensitivity C-reactive protein (hsCRP), were tested, and *Kt*/*v* were calculated for all patients.

(2) Blood pressure was recorded for all patients before and after hemodialysis at first hemodialysis of every season. Average value, standard deviation, and variation coefficient of SBP and DBP, average arterial pressure, ΔSBP[ΔSBP = SBP(before dialysis)−SBP(after dialysis)] and ΔMBP[ΔMBP = MAP(before dialysis)−MAP(after dialysis)] were also calculated.

Patients were divided into four groups, based on their relative ΔSBP level: Group I: first quartile (ΔSBP < P_25_); Group II: second quartile (P_25_≤ΔSBP < P_50_); Group III: third quartile (P_50_≤ΔSBP < P_75_); and Group IV: fourth quartile (ΔSBP ≥ P_75_). General conditions of patients in the four groups were compared with each other.

### Statistical methods

SPSS (version 20.0) software (SPSS Inc., Chicago, IL) was used for statistical analysis. Normal distributed measurement data were represented by mean ± standard deviation, and the Bonferroni test was used for pairwise comparisons among groups. Measurement data that were not normally distributed were represented by median and quartile, and the Mann–Whitney *U* test was adopted for comparisons between groups. Comparisons between groups for quantitative data were conducted using the Chi-square test or the Fisher exact test. Cox regression analysis was applied to analyze risk factors for all-cause mortality for MHD patients. Kaplan–Meier survival curve was used to analyze the relationship between ΔSBP and survival rate in MHD patients.

## Results

### Clinical manifestations and biochemical tests

Three hundred and sixty-four patients, 221 (60.7%) male, with an average age of 63.07 ± 13.93 years and an average dialysis vintage of 76.00 (range, 42.25–134.00) months, were selected. There were 85 cases (23.4%) of all-cause death and 46 cases (14.2%) of cardiovascular death (see Table 1 for patients’ demographic data and biochemical test results).

**Table 1.** Basic patient demographic characteristics and laboratory data, classified by quartiles of ΔSBP.

		Quartiles of ΔSBP (mmHg)			
	All (*n* = 364)	ΔSBP < −0.25	−0.25 ≤ ΔSBP < 7.09	7.09 ≤ ΔSBP < 14.25	ΔSBP ≥ 14.25
Number (*n*, %)	364 (100%)	92 (25.3)	90 (24.7)	91 (25.0)	91 (25.0)
Male, *n* (%)	220 (60.6)	61 (27.7)	57 (63.3)	56 (61.5)	46 (51.1)
Age (years)	63.07 ± 13.93	65.02 ± 13.72	62.47 ± 15.82	61.11 ± 11.95	63.50 ± 13.99
Dialysis vintage, (month)	76.00 (42.25–134.00)	59.50 (32.0–96.5)	72.00 (50.75–124.5)^b^	81.00 (46.0–144.0)^b^	105.5 (48.75–144.25)^b^
Follow up (month)	54.86 ± 19.84	48.99 ± 21.85	56.72 ± 18.14^b^	57.89 ± 18.27^b^	56.48 ± 19.40^b^
BMI (kg/m^2^)	22.06 ± 3.88	21.15 ± 4.05	21.77 ± 4.01	22.53 ± 3.55	22.83 ± 3.77^b^
*Kt*/*v*	1.71 (1.50–1.99)	1.70 (1.48–2.03)	1.74 (1.49–1.91)	1.66 (1.47–1.95)	1.75 (1.58–2.03)
Ca (mmol/l)	2.33 (2.21–2.46)	2.29 (2.17–2.39)	2.32 (2.19–2.41)	2.34 (2.23–2.48)	2.41 (2.24–2.56)^b^
P (mmol/l)	1.93 ± 0.40	1.87 ± 0.39	1.90 ± 0.40	1.97 ± 0.39	2.00 ± 0.42^b^
IPTH (pg/ml)	307.43 (157.20–536.09)	225.00 (141.10–376.00)	317.83 (137.32–539.34)^b^	367.17 (153.00–630.86)^b^	357.50 (235.56–585.12)^b^
hsCRP (mg/l)	17.13 (7.49–36.05)	14.37 (4.76–30.88)	16.34 (7.62–42.24)	20.23 (6.85–33.95)	20.33 (9.70–42.25)
Alb (g/l)	39.35 ± 3.07	39.28 ± 3.42	39.36 ± 3.08	39.63 ± 2.73	39.28 ± 3.04
Hb (g/l)	107.89 ± 11.40	106.22 ± 12.21	107.83 ± 9.55	109.74 ± 12.66^a^	107.90 ± 10.78
Triglycerides (mmol/l)	1.78 ± 0.86	1.79 ± 0.96	1.78 ± 0.79	1.78 ± 0.76	1.79 ± 0.94
Total cholesterol (mmol/l)	4.34 ± 0.76	4.31 ± 0.85	4.36 ± 0.69	4.32 ± 0.79	4.35 ± 0.82
Low-density lipoprotein (mmol/l)	2.35 ± 0.79	2.37 ± 0.67	2.34 ± 0.82	2.35 ± 0.68	2.36 ± 0.84
High-density lipoprotein (mmol/l)	1.17 ± 0.63	1.15 ± 0.59	1.19 ± 0.68	1.16 ± 0.58	1.18 ± 0.66
Anti-hypertensive					
ACEI/ARB (*n*, %)	196 (53.8)	46 (50.0)	52 (57.8)	50 (54.9)	48 (52.7)
CCB (*n*, %)	165 (45.3)	44 (47.8)	38 (42.2)	41 (45.1)	42 (46.2)
α,β-block (*n*, %)	72 (19.8)	18 (19.6)	16 (17.8)	21 (23.1)	17 (18.7)
Mortality					
All cause (*n*, %)	84 (23.1)	33 (35.9)	16 (17.8)^a^	13 (14.3)^b^	22 (24.4)
Cardiovascular (*n*, %)	46 (12.7)	17 (18.5)	11 (12.2)	6 (6.6)^a^	12 (13.3)

^a^Compares with ΔSBP< −0.25 group, *p* < .05.

^b^Compared with ΔSBP< −0.25 group, *p* < .01.

Average SBP and DBP and MBP after dialysis were all higher in Group I compared with the other three groups; however, there was no statistically significant difference regarding average pre-dialysis SBP, DBP, SBP CV, and post-dialysis SBP SD among the four groups (see Table 2 for differences in blood pressure before and after dialysis).

**Table 2.** Blood pressure changes before and after dialysis in maintenance hemodialysis patients.

		Quartiles of ΔSBP (mmHg)			
	All (n = 364)	ΔSBP < −0.25	−0.25 ≤ ΔSBP < 7.09	7.09 ≤ ΔSBP < 14.25	ΔSBP ≥ 14.25
Before Dialysis					
SBP SD (mmHg)	18.67 ± 7.4	19.52 ± 9.21	17.19 ± 6.07^a^	18.51 ± 6.61	19.47 ± 7.2
Average SBP (mmHg)	142.49 ± 18.4	141.32 ± 19.22	139.48 ± 18.26	142.89 ± 15.95	146.09 ± 19.64
SBP CV	0.133 ± 0.054	0.139 ± 0.065	0.124 ± 0.440	0.131 ± 0.050	0.136 ± 0.054
DBP SD (mmHg)	10.19 ± 3.56	10.28 ± 4.03	9.84 ± 3.22	10.52 ± 3.70	10.16 ± 3.25
Average DBP (mmHg)	76.69 ± 10.65	76.46 ± 11.87	75.25 ± 10.99	77.52 ± 8.78	77.52 ± 10.77
DBP CV	0.136 ± 0.052	0.137 ± 0.059	0.134 ± 0.048	0.138 ± 0.054	0.133 ± 0.048
MBP (mmHg)	98.62 ± 12.00	98.08 ± 13.16	96.66 ± 12.13	99.31 ± 9.92	100.38 ± 12.48
After Dialysis					
SBP SD (mmHg)	19.79 ± 7.74	20.57 ± 9.47	18.77 ± 7.28	20.21 ± 7.00	19.18 ± 6.89
Average SBP (mmHg)	135.09 ± 19.67	147.88 ± 19.23	136.16 ± 18.06^b^	131.74 ± 16.14^b^	124.34 ± 17.53^b^
SBP CV	0.148 ± 0.056	0.140 ± 0.063	0.138 ± 0.051	0.154 ± 0.052	0.159 ± 0.055^a^
DBP SD (mmHg)	10.69 ± 4.13	10.77 ± 4.86	9.82 ± 4.55	10.98 ± 3.33	11.16 ± 3.49
Average DBP (mmHg)	74.84 ± 10.96	78.92 ± 11.71	75.79 ± 10.86^a^	74.72 ± 9.03^b^	69.84 ± 10.25^b^
DBP CV	0.145 ± 0.057	0.138 ± 0.062	0.132 ± 0.062	0.150 ± 0.051	0.161 ± 0.050^b^
MBP (mmHg)	94.93 ± 12.79	101.91 ± 13.02	95.91 ± 12.16^b^	93.73 ± 10.40^b^	88.01 ± 11.56^b^

^a^Compares with ΔSBP < −0.25 group, *p* < .05.

^b^Compared with ΔSBP < −0.25 group, *p* < .01.

### The relationship between ΔSBP before and after hemodialysis and risk of all-cause and cardiovascular death

Compared with patients in Group I (ΔSBP< −0.25 mmHg), the risk of all-cause death for patients in Group II (−0.25 mmHg ≤ ΔSBP <7.09 mmHg) and Group III (7.09 mmHg ≤ ΔSBP <14.25 mmHg) was significantly lower. After correcting for age, gender, dialysis age, general individual conditions, and biochemical indices, the difference was still statistically significant. For patients in Group IV (ΔSBP ≥14.25 mmHg), the risk of all-cause death decreased by 25.2% compared with Group I (ΔSBP< −0.25 mmHg), but there was no statistically significant difference between the two groups (see Table 3). Among the patients who died of cardiovascular diseases, the risk of cardiovascular death for patients in Group III (7.09 mmHg ≤ ΔSBP< 14.25 mmHg) was significantly lower than for those in Group I (ΔSBP< −0.25 mmHg), and this difference remained statistically significant even after correcting for all potential confounding factors (see Table 4).

**Table 3.** Association of ΔSBP and all-cause mortality.

	Quartiles of ΔSBP (mmHg)			
	I ΔSBP < −0.25	II −0.25 ≤ ΔSBP < 7.09	III 7.09 ≤ ΔSBP < 14.25	IV ΔSBP ≥ 14.25
*n* with death/total	33/92	16/90	13/91	22/91
unadjusted	1	0.387 (0.194–0.769) *p* = .007	0.298 (0.144–0.616) *p* = .001	0.578 (0.304–1.100) *p* = .095
Fully adjusted^a^	1	0.421 (0.199–0.888) *p* = .023	0.324 (0.152–0.692) *p* = .004	0.748 (0.366–1.529) *p* = .426

^a^Adjusted for age, sex, dialysis vintage, calcium, phosphorus, hemoglobin, hsCRP, *Kt*/*v*.

**Table 4.** Association of ΔSBP and cardiovascular disease mortality.

	Quartiles of ΔSBP (mmHg)			
	I ΔSBP < −0.25	II −0.25 ≤ ΔSBP < 7.09	III 7.09 ≤ ΔSBP < 14.25	IV ΔSBP ≥ 14.25
*n* with death/total	17 (92)	11 (90)	6 (91)	12 (91)
unadjusted	1	0.614 (0.270–1.397) *p* = .245	0.311 (0.117–0.831) *p* = .020	0.679 (0.304–1.517) *p* = .345
Fully adjusted^a^	1	0.483 (0.190–1.228) *p* = .126	0.335 (0.123–0.911) *p* = .032	0.435 (0.160–1.181) *p* = .435

^a^Adjusted for age, sex, dialysis vintage, calcium, phosphorus, hemoglobin, hsCRP, *Kt*/*v*.

### Analysis of risk factors for all-cause mortality in MHD patients

The primary outcome of the study was death from any causes (all-cause mortality). COX regression analysis showed that the following factors were independently associated with all-cause mortality in MHD patients: ΔSBP> −0.25 mmHg (OR = 0.472, 95%CI = 0.302–0.738, *p* = .001); hemoglobin concentration (OR = 0.575, 95%CI = 0.461–0.716, *p* < .001); and *Kt*/*v* (OR = 0.439, 95%CI = 0.260–0.739, *p* = .002); albumin (OR = 0.397, 95%CI = 0.242–0.650, *p* < .001).

### Relationship between ΔSBP and survival rate

**Figure 1.**
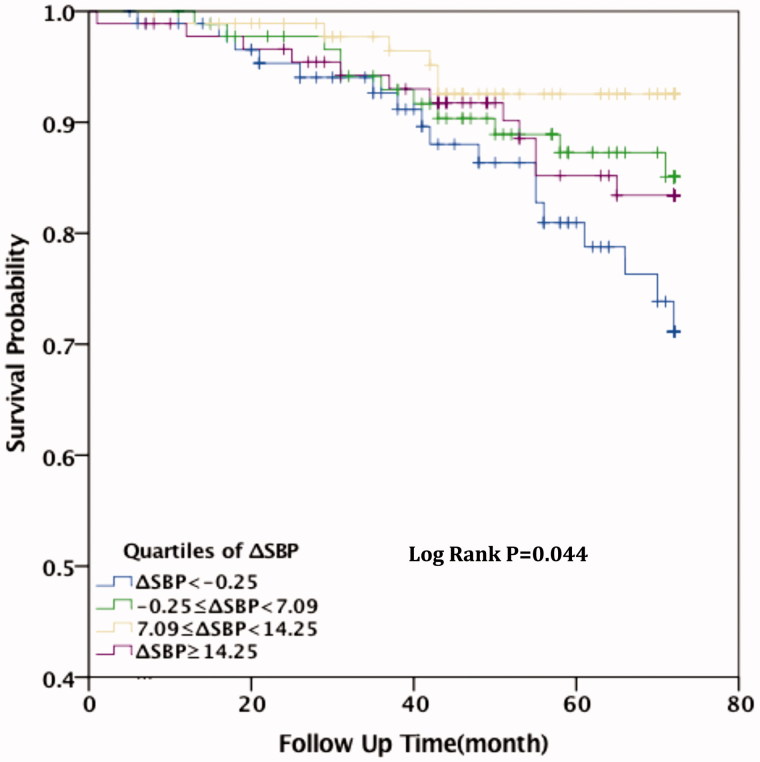
Kaplan–Meier shows the relationship between ΔSBP and all-cause mortality.

**Figure 2.**
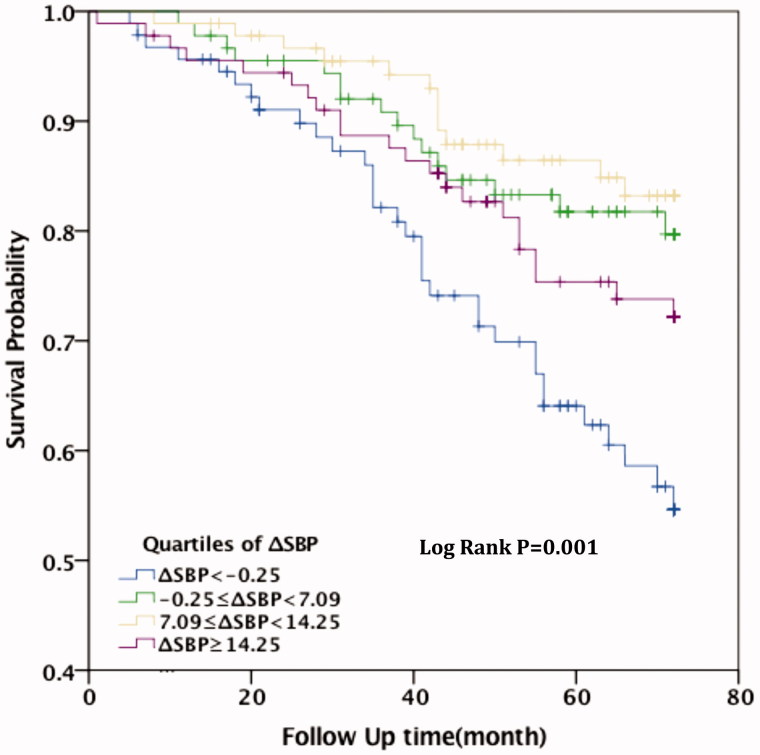
Kaplan–Meier shows the relationship between ΔSBP and cardiovascular disease mortality.

Kaplan–Meier analysis showed that, for patients with ΔSBP < −0.25 mmHg, all-cause mortality and cardiovascular mortality were all significantly higher than for those in the other three groups (*p* = .001 and .044, respectively). Compared with patients in Group I (ΔSBP < −0.25 mmHg), the survival rate of cardiovascular death for patients in Group II (−0.25 mmHg ≤ ΔSBP <7.09 mmHg), Group III (7.09 mmHg ≤ ΔSBP <14.25 mmHg), and Group IV (ΔSBP ≥14.25 mmHg)was significantly higher (*p* = .003, *p* < .001, and *p* = .039, respectively). Among the patients who died of all-cause death, the survival rate was higher in Group III (7.09 mmHg < ΔSBP <14.25 mmHg) compared with Group I (ΔSBP < −0.25 mmHg) (*p* = .005). The difference was no statistically significant among Group II, Group IV, and Group I (*p* = .109 and .171, respectively). This indicates that patients with higher SBP after hemodialysis had lower survival rate (see Figures 1 and 2).

## Discussion

Blood pressure increase or decrease frequently occurs among patients during hemodialysis. Physicians usually pay attention to the development of hypotension and other complications occurring during hemodialysis, while the significance of blood pressure increase or moderate decrease during dialysis is easily overlooked. In this study, 364 MHD patients were investigated, and the results showed that patients with ΔSBP< −0.25 mmHg during dialysis had higher mortality, while those patients whose SBP moderately decreased had a higher survival rate.

The increase in SBP during hemodialysis was closely related to the increase in mortality, and SBP is increased in about 10–15% of patients after dialysis, according to some studies.^8^ Previous studies have also shown that SBP increase during hemodialysis is a sign of poor prognosis in the short term.^9^ However, other studies have found that increased SBP variability in MHD patients before dialysis was an independent risk factor for all-cause mortality.^7^ In our study, there was no statistical difference in average pre-dialysis SBP or coefficient of SBP variation for patients whose SBP increased > −0.25 mmHg after dialysis, compared with the other three groups. In this study, the increased risk of all-cause mortality caused by an increase in SBP after dialysis was not affected by the average pre-dialysis SBP value and by SBP variability.

Blood pressure after dialysis compared with blood pressure before dialysis can be used to estimate cardiac stress during dialysis.^10^ In addition, the relationship between the increase in blood pressure during dialysis and poor outcomes reflects an increase in cyclic pressure loads.^11^^,^^12^ In the present study, hypertension related to dialysis might have been caused by excessive volume load, since many MHD patients did not reach their dry weight, and ultrafiltration of excessive fluid for patients during dialysis could have increased cardiac output, thereby increasing blood pressure, possibly caused by an altered Frank–Starling curve.^13^ However, other studies have indicated that endothelial dysfunction may be an important factor for the blood pressure increase occurring during dialysis. These studies have found that, for patients whose blood pressure increased during dialysis, peripheral vascular resistance also increased after dialysis,^14^ which was not related to the secretion of catecholamines and renin, but was related to endothelin-1 and nitric oxide. Some researchers have suggested that blood pressure increase during dialysis may be related to endothelial cell dysfunction. Endothelial cell injury is a sign of vascular injury, and it could increase the risk of death due to cardiovascular disease.^15–17^ In the present study, we found that, for patients whose blood pressure increase was >0.25 mmHg after dialysis, cardiovascular mortality was also significantly increased, the mechanism of which could involve endothelial cell dysfunction.

Large decreases in SBP or DBP during hemodialysis can also increase mortality, while patients with moderate decreases have a higher survival rate, according to previous studies.^18^ Hypotension during dialysis is a common complication, and patients whose SBP decreases during dialysis and for whom orthostatic hypotension occurs after dialysis also demonstrate significantly decreased survival.^19^ For patients without coronary artery disease, a decrease in left ventricular function can be caused by myocardial ischemia resulting from coronary blood flow reduction^20^ or stressors on blood vessels during hemodialysis, etc. These factors together can cause irreversible damage to the heart.^21^^,^^22^ Thus, a large decrease in blood pressure during hemodialysis may damage the cardiovascular system of patients, resulting in increased mortality. This study has found that patients with ΔSBP >14.25 mmHg did not have higher survival or lower cardiovascular mortality compared with those with ΔSBP< −0.25 mmHg, and there was no significant difference in their survival rate, indicating that large decreases of SBP after dialysis did not benefit patients’ survival and may increase their risk of death.

However, this study has certain limitations. First of all, it analyzed the relationship between survival and blood pressure decrease after dialysis, the extent of blood pressure decrease, and blood pressure increase after dialysis. Since the lowest blood pressure of patients might be lower than the blood pressure measured immediately after dialysis, the blood pressure value after dialysis may not accurately represent the impact of the lowest blood pressure during dialysis on patients’ survival. Moreover, blood pressure after dialysis might be influenced by multiple potential confounders. In addition, the use of antihypertensive drugs was not taken into account in this study, and the potential effects of various antihypertensive drugs on patient survival were not studied. Furthermore, this study did not consider the impact of residual renal function.^23^ Residual renal function could be independently related to mortality in MHD patients, since it contributes to the relative stability of volume and could decrease weight fluctuations during dialysis. Moreover, the exact pathophysiology of blood pressure fluctuations during hemodialysis has not been identified in this study, and the impact of various factors (e.g. ultrafiltration method, temperature of dialysis fluid, etc.) on patients’ blood pressure fluctuations were unknown. Finally, this was a retrospective observational study. Patients were not randomized, and only those confounders were corrected for that had been recognized, detected, and measured.

To summarize, SBP changes before and after dialysis in MHD patients were closely related to both all-cause and cardiovascular mortality. Patients with moderate decreases of blood pressure after dialysis had a higher survival rate, while patients whose blood pressure decreased less than 0.25 mmHg or increased after dialysis compared with that before dialysis demonstrated significantly decreased rates of survival. Consequently, blood pressure changes occurring during dialysis could be used as a treatment target to improve the prognosis for MHD patients. However, the results of this study should be verified by prospective, randomized, controlled studies involving a large number of patients.
